# Remembering a name: Neuropsychological validity studies and a computer proposal for detection of anomia

**DOI:** 10.1590/1980-57642018dn13-040013

**Published:** 2019

**Authors:** Nora Silvana Vigliecca, Javier Alfredo Voos

**Affiliations:** 1 Servicio de Neurología y Neurocirugía del Hospital Córdoba, Argentina.; 2 Universidad Tecnológica Nacional (UTN), Regional Córdoba; Córdoba, Argentina.

**Keywords:** Alzheimer’s disease, anomia, cognitive impairment, diagnosis, neuropsychological tests, reliability and validity, doença de Alzheimer, anomia, comprometimento cognitivo, diagnóstico, testes neuropsicológicos, confiabilidade e validade

## Abstract

There are contradictory results or lack of validity studies concerning the naming function and brain laterality. Although anomia is a frequent symptom of memory impairment, and the most relevant symptom of aphasia, few studies have been conducted to evaluate its validity for detecting patients with left-hemisphere damage (LD), as per the MeSH definition.

**Objective:**

To validate a paper-and-pencil confrontation naming test (CNT) according to side of brain injury; to select a valid and reliable abbreviated CNT wherein the effect of demographic variables is minimized; and to use the selected CNT to develop a computer-aided confrontation-naming evaluation (CACNE).

**Methods:**

Control data were obtained from 213 healthy participants (HP) aged 15 to 89 years. A subsample of 106 HP was demographically matched to 39 patients with LD and 40 patients with right-hemisphere damage (RD). Anomia definition and CNT cues were considered for the CACNE.

**Results:**

Test-retest and inter-rater reliability, internal consistency, and validity for detecting LD were demonstrated. A significant age effect was observed in HP. The CACNE was developed to detect anomia in interaction with environmental interventions.

**Conclusion:**

The inconsistencies observed in the CNT studies were probably due to the presence of anomia in almost 50% of the RD patients.

## RESUMO

Existem resultados contraditórios ou falta de estudos de validade relativos à função de nomeação e lateralidade cerebral. Embora a anomia seja um sintoma frequente de comprometimento da memória e o sintoma mais relevante da afasia, poucos estudos foram realizados para avaliar sua validade na detecção de pacientes com lesão no hemisfério esquerdo (LHE) conforme definição do MeSH.

**Objetivo:**

Validar um teste de nomeação por confrontação (TNC) do tipo papel-e-lápis de acordo com o lado da lesão cerebral. Selecionar um TNC abreviada válida e confiável, na qual o efeito de variáveis ​​demográficas seja minimizado. Usar o TNC selecionado para desenvolver uma avaliação de nomeação por confrontação auxiliada por computador (CACNE).

**Métodos:**

Os dados de controle foram obtidos de 213 participantes saudáveis ​​(PS) com idades entre 15 e 89 anos. Uma subamostra de 106 PS foi demograficamente pareada com 39 LHE e 40 pacientes com lesão no hemisfério direito (LHD). A definição de anomia e as pistas do TNC foram consideradas para a CACNE.

**Resultados:**

A confiabilidade teste-reteste e interexaminador, consistência interna e validade para a detecção de LHE foram demonstradas. Um efeito significativo da idade foi observado na PS. CACNE foi desenvolvida para detectar anomia em interação com intervenções ambientais.

**Conclusão:**

As inconsistências observadas nos estudos do TNC devem-se provavelmente à presença de anomia em quase 50% dos pacientes com LHD.

According to the MeSH, anomia is defined as “A language dysfunction characterized by the inability to name people and objects that are correctly perceived. The individual is able to describe the object in question, but cannot provide the name. This condition is associated with lesions of the dominant hemisphere…”.^1^ [Note: in this study we will only refer to the inability to name objects, not people].^1^

Although anomia is considered a common early sign in primary progressive aphasia (PPA)^2^ and the most relevant symptom of acute aphasia,^3^ it is also considered a common symptom in most types of dementia and non-focal brain disease.^2^^,^ 4 ^-^ 6 Milder forms of anomia are among the most common complaints in normal aging^5^ and, when memory is affected, anomia is one of the most frequent symptoms associated with the underlying syndrome. 

Likely based on the above assumptions, and in spite of the definition of anomia, few studies have been conducted to evaluate the validity of this measure for detecting lesions in the left hemisphere. In any case, contradictory findings have been reported when confrontation naming tests (CNT), in particular, have been employed (see reports).^7^^-^ 13

Regarding the influence of demographic variables on these tests, results are also contradictory and highly dependent on participant recruitment.^14^

Most of the validity studies based on CNT have been carried out in patients with Alzheimer’s disease (AD) and, within this framework, clinical studies indicate that AD disproportionately affects women in terms of both disease prevalence and rate of symptom progression.^15^^,^ 16 However, little work has been done to determine the validity of the relationship among gender (or demographic variables in general), aging, and anomia.

Naming tests can be considered language indicators of semantic memory: in visual CNT, the interviewee is usually assessed through pictures, in which he/she must recognize their function or meaning, involving visual knowledge, and retrieve their names, involving verbal knowledge.^17^ Although a correlation between naming and cognitive impairment has been observed,^2^^-^ 6 ^,^ 17 questions remains regarding the validity of the CNT total score for the detection of anomia in some disorders which supposedly have this symptom (see papers).^9^^,^ 12 ^,^ 18

Considering brain laterality and naming, some new perspectives have emerged (focused mainly on specific brain areas or pathologies), and there is now a substantial body of work about this issue.^12^^,^ 13 ^,^ 19 ^-^ 28 Despite using a different approach to the one employed in the present study, these perspectives aid understanding and encourage validity studies. For example, there are a number of areas involved in aspects of language comprehension and production in both hemispheres and neuroimaging results suggest that various cognitive tasks that make use of similar representations or processes frequently share component sub-functions with other tasks including non-linguistic ones.^25^ The interaction between different inputs and outputs,^13^ and the dissociation between left brain areas for lexical retrieval and right brain areas for visual recognition,^26^ have been reported in these perspectives.

Semantic memory has been linked to the anterior temporal lobes (ATLs) in both hemispheres, in patients with temporal lobe epilepsy (TLE).^13^ Alternatively, semantic verbal memory in particular, has also been linked to the inferior part of the left temporal lobe^12^^,^ 27 in patients with TLE, as well as to the tip of the left ATL in patients with PPA.^26^

A previous fMRI meta-analysis showed that visual semantic concept tasks exhibit strong bilateral activation across the ATLs in healthy participants.^28^ In addition, the speech production system in the prefrontal cortex has been shown to be highly left lateralized in neuroimaging studies, i.e. the left ATL seems to be more strongly connected to regions involved in speech production than the right, and thus is likely to assume some specialization for naming tasks.^13^ Miozzo and Hamberger,^11^ suggested that when picture meaning is preserved, the probable cause of word-finding difficulty in the left TLE relates to processes that involve lexical/phonological information.

These views are relevant for the present study because the current CNT deals with both linguistic and non-linguistic stimuli, as well as with the concept of semantic cognition. 

The present study involves a CNT which was initially based on that of Oldfield and Wingfield,^29^ which had validity studies associated with lesions of the verbal dominant hemisphere,^8^ but their results were obtained from a sample of men only. In a preceding study with this test and a sample of healthy participants (HP) aged 9 to 90 years,^14^ women of greater age and lower education level were mainly affected in their naming performance. Although these results are in agreement with findings observed in AD, further study is necessary to ascertain whether they were associated with some artifactual effect, such as the sample of participants or items selected or, considering that AD involves both sides of the brain, the contribution of one of these sides in particular. The study of unilateral focal brain lesions may help elucidate this issue.

Given the lack of validity studies and presence of contradictory results with the naming function regarding brain laterality, the main objective of the present study was to validate a CNT according to the side of brain injury.

Since the present study is part of a bigger research project developing brief, efficient and/or easy-to-apply neuropsychological techniques, the complementary objectives of the present study were: 

To abbreviate the initial CNT from 60 to 30 items by developing two parallel forms from the test and selecting one of them.

To verify the internal consistency and reliability of the abbreviated scales and how they were influenced by demographic variables

To verify the validity of the abbreviated scales to discriminate patients with left hemisphere damage and, if possible, select a valid and reliable abbreviated scale wherein the effect of demographic variables is minimized. 

To use the selected abbreviated scale to develop a computer-aided confrontation-naming evaluation (CACNE).

## METHODS

### Material

We used the CNT from the battery of Neuropsychological Tests Abbreviated and Adapted to Spanish Speakers (NTAASS).^30^^-^ 33 This paper-and pencil-CNT, with black-and-white drawings, consists of 60 items, of which 36 were translated and adapted from the Oldfield and Wingfield’s scale.^29^

Since in a previous study (using 45 items instead of the original 36), a gender effect was observed which was significant from the age of 60 years for any level of education,^14^ we predominantly recruited participants in this age range for the present study. Also, the number of items was increased from 45 to 60 to create two abbreviated and parallel forms of 30 items, and select one of these forms to develop a simpler evaluation of anomia. The last 15 items of the extended scale were designed to be as neutral as possible to the effect of demographic variables (e.g.to avoid an unintentional biased selection of items favoring males).^14^^,^ 34

Since the overlapping effects of stimulus characteristics (frequency, acquisition age, decline age, etc.) are ultimately related to accuracy (i.e. item difficulty), we first used face validity to avoid, e.g. the selection of items favoring males, and then the effect of each item on the scales was verified case-by-case, according to the demographic variables for this particular sample and context. We also added items of lower frequency, such as geometric figures, with the intention of achieving a wide range of difficulty. Regarding the age at acquisition, it is worth noting that picture naming is a current approach to establish this word property, which relates to familiarity, frequency of use, etc. (see articles^35^^,^ 36 and below the procedure for parallel forms, where the item-difficulty approach was also used).

We used the Oldfield and Wingfield’s scale because the battery of the NTAASS^30^^-^ 33 was developed in around 1998 when validity studies were even scarcer. Thus, this CNT was selected in view of its validity studies regarding both lesion side^8^ and the relationship between item frequency and response speed.^29^^,^ 34 Moreover, this test was taken as a reference to develop other naming tests. Regarding our research resources, we tried to improve this test in particular, because of our previous experience with its content and the material collected.

### Participants and procedures

Data were obtained from a sample of 292 Argentine right-handed volunteers, who were all native Spanish speakers. 

Control data were obtained from 213 healthy participants (HP), who were community-dwellers, without any known neurological or psychiatric disease. The recruitment method is further described elsewhere.^30^^,^ 31 ^,^ 37 ^-^ 40

Clinical data were obtained from 79 consecutive preoperative inpatients with focal and unilateral cerebral lesions. The sample was recruited from the Neurological and Neurosurgery Service of the Cordoba Hospital, a public hospital for adults. Lesions were confirmed by CT scan and/or MRI techniques, as well as by complementary diagnostic studies. None of the patients suffered from any other (previous or simultaneous) associated neurological disease. 

We excluded patients who were unable to understand the test instructions or who had visual agnosia, hemianopia, hemineglect, or sensory or motor difficulties which might affect visual perception. We also excluded patients who answered less than 50% of the first ten pictures of the initial scale correctly,^14^ or who had a severe impairment in describing the pictures when anomia was present (i.e. patients were excluded if they had unintelligible or severely distorted expressions on more than 15% of all pictures).

The initial sample consisted of 83 patients, from which one patient was excluded for probable visual agnosia, another for having hemianopia with hemineglect, a third one because of difficulty understanding the test instructions and answering less than 50% of the first ten pictures of the initial scale correctly; finally, another patient was excluded for producing severely distorted descriptions on more than nine pictures of the initial scale. 

We excluded patients with visual agnosia or with visual-perception difficulties, because we sought to assess genuine word retrieval failures or anomic states.^12^ As we assessed word retrieval in the spontaneous performance of the CNT, which was the first participants’ response, if participants answered with only a description of the object, this response was considered wrong (see statistical analysis below and also the CACNE for an analysis of this failure using an approach in which the description of the object is computed using visual and auditory inputs).

As we needed interviewees to recognize CNT pictures, when the presence of conceptual blurring was suspected (i.e. disruptions in single-word association and comprehension^26^ or in visual-perception) we administered the optional card from the Brief Aphasia Evaluation.^3^^,^ 30 ^,^ 31 ^,^ 37 ^,^ 38 In this card, not only can real objects be matched with pictures of the same objects (picture-picture recognition), but also interviewers can say the words of the pictures on the card while interviewees point at the corresponding picture (word-picture recognition). Interestingly, this card includes pictures of the same family of objects (e.g. pen, pencil; matches, lighter; car key, house key) and/or semantically associated representations (e.g. remote control for car key; school accessories for pen and pencil, etc.), both of which could produce semantic interference.^26^

Regarding clinical intervening variables, no significant differences were observed between patients with left and right-hemisphere damage (LD and RD, respectively) for disease duration (mean months: LD: 33.44±82.72, RD: 34.05±84.99 (F(1,77)=0.00, p=0.97); risk factors for cognitive impairment (mean number: LD: 1.28±1.12, RD: 1.42±1.17 (F(1,77)=0.30, p=0.58); site of lesion (frequency: anterior (frontal): LD=9, RD=13, posterior (temporal, parietal or occipital): LD=19, RD=19, antero-posterior: LD=5, RD=5, and subcortical: LD=6, RD=3 (Chi^2^=1.71; df: 3; p=0.63)); and type of lesion (see Table 1 for specific types, and for the difference between malignant tumors and rest of lesions, which numbered 23 for LD and 26 for RD (Chi^2^=0.30; df: 1; p=0.58)); no significant differences were found when specific lobe lesions were also compared (results available on request). 

**Table 1:** Frequency of focal brain lesions by type and patient group.

Lesion Type	Group	
	LD	RD
AVM	4	5
BEN TU	9	8
MAL TU	16	14
ISQ STR	1	3
HEM STR	4	4
TBI	2	2
Other	3	4
Total	39	40
Chi^2^ = 1.43; df: 6; p=0.96		

LD: patients with left hemisphere damage, RD: patients with right hemisphere damage; AVM: Arteriovenous malformation, BEN TU: Benign tumor, MAL TU: Malignant tumor, ISQ STR. Ischemic stroke, HEM STR: Hemorrhagic stroke, TBI traumatic brain injury, OTHER: cyst, mesial temporal sclerosis, aneurysm, abscess (LD: one case for each type, but no case for cyst; RD: one case for each type). Chi2: Chi-square statistics; df: degrees of freedom; p: p-value.

For entry to this study, all participants (or their caregivers) signed the informed consent form. The pertinent institutional review board approved the project and the study, which was carried out in accordance with the ethical standards established in the Declaration of Helsinki.^41^

We developed the parallel forms from the initial CNT and verified the internal consistency of these abbreviated scales, along with the effect of demographic variables in a sample of 213 HP (see Table 2) aged 15 to 89 (mean±SD: 54.96±19.38) years. 

**Table 2:** HP - sample demographic data.

Gender	Age range	Education			Row totals
		First level	Second level	Third level	
F	15-29	10	5	4	19
F	30-44	11	4	2	17
F	45-59	13	5	6	24
F	60-74	32	14	6	52
F	75-90	13	6	1	20
Total		79	34	19	132
M	15-29	9	4	3	16
M	30-44	7	2	2	11
M	45-59	9	4	3	16
M	60-74	15	8	5	28
M	75-90	6	3	1	10
Total		46	21	14	81
Column Total		125	55	33	213

HP: healthy participants; F: female; M: male.

From this total sample, a sub group of 37 participants was re-evaluated for test-retest and inter-rater reliability. In this subsample of 17 to 84-year-olds (45.41±20.66), 62% (N=23) of the participants were females and 38% (14) males; 40% (15) had first level education, 38% (14) second level and 22% (8) third level. 

In this study, all the participants, either HP or patients, received the original 60 items (the initial scale) at the time of testing in any step of the research. With the data provided by the participants, we then developed the abbreviated scales by analyzing and modeling two parallel forms and selecting one of them. Subsequently, the CACNE was developed using the selected scale.

We developed the two parallel forms of the initial scale (i.e., form 1 (F1) and form 2 (F2)) by verifying that they were comparable in terms of difficulty and relationship with demographic variables. The parallel forms were selected by considering the items of the initial scale which contributed most to attenuate both demographic differences in general, and the effect of gender and its interactions in particular.^14^ Items with these properties were distributed between the scales in an effort to achieve parallelism and the study objectives. The selection of one of the scales was finally based on the fulfillment of most of the criteria that arose from the different analyses of this study (including studies of demographic variables, validity and reliability), with particular emphasis on the criterion for side of brain lesion (see below). 

For test-retest reliability, at least 30 days of inter-test interval was established, with a maximum of 120 days. For inter-rater reliability, two trained interviewers acting independently evaluated the spontaneous (uncued) performance of the same interviewee. Specifically, the second rater interpreted the first rater’s written records of the administration protocol, in which multiple variables were registered.

A subsample of HP was matched with LD and RD, according to demographic variables (see Table 3). With these subjects we determined the validity of the scales for discriminating between non-LD (RD and HP) and LD on the one hand, and among LD, RD and HP on the other hand. 

**Table 3:** Demographic data for the three matched samples.

Group	Age (mean ±SD)	Education (three-level frequency)	Gender (male frequency)	N
LD	41.26±16.83	22 14 3	22	39
RD	43.67±13.01	22 16 2	23	40
HP	42.19±15.81	67 30 9	48	106
Total	42.31±15.42	111 60 14	93	185
	F(2,182)=0.25	Chi^2^=2.31; df:4	Chi^2^=2.48; df:2	
	p=0.78	p=0.68	p=0.29	

LD: patients with left hemisphere damage; RD: patients with right hemisphere damage; HP: healthy participants. F-statistics with degrees of freedom; Chi^2^: Chi-square statistics; df: degrees of freedom; p: p-value.

In developing the CACNE, we took into account the definition of anomia^1^ and the cues usually implemented to help remedy anomia. To this end, previous findings about CNT were reviewed,^42^^-^ 46 particularly those regarding the type of performance errors (see articles).^47^^-^ 49 We make available the CACNE as a free CNT to be tested using researchers’ particular samples and objectives. 

### Statistical analysis

The correlation between the two parallel forms, and of these forms with the initial CNT, was analyzed by the Pearson correlation coefficient (r). The internal consistency for either F1 or F2 was analyzed by Cronbach’s alpha coefficient, while the difference between the two forms was explored by - Student’s *t*-test for dependent samples.

A gender × age × education ANOVA was carried out to determine the effect of demographic variables on the spontaneous performance of CNT, which was the number of correct responses (correct=1, error=0) on any of the three scales. Age was recoded into five levels (see Table 2); education was recoded into two levels (first level versus higher levels) to avoid the presence of empty cells and/or lack of variance, particularly for the third level of education. 

Test-retest and inter-rater reliability indices were analyzed by the intra-class correlation coefficient (ICC). Differences between test and retest and differences between both raters were analyzed by Student’s *t*-test for dependent samples.

When HP were compared with LD and RD for demographics, data were analyzed by ANOVA for years of age or by Chi^2^ for education and gender.

Receiver Operating Characteristic (ROC) curve analysis was performed to determine the sensitivity and specificity of the scales for differentiating non-LD and LD. The cut-off point that produced a more uniform frequency distribution between sensitivity and specificity was considered the most satisfactory. When this was not possible, or when one of the frequencies was <70% (see below), sensitivity was prioritized. In order to select optimal cut-off points based on several criteria, ROC curve analysis was done with the “Statistica” computer program, using neural networks and a linear model.^50^

The validity of the scales for discriminating among LD, RD and HP was verified by cross-tabulation and Chi^2^, using the selected cut-off points. 

In general, indices ≥70% (correlation, reliability, validity, etc.) were considered acceptable.

## RESULTS

The r between F1 and F2 was 0.81 and between either of these forms and the initial CNT was 0.95 (N=213). The Cronbach’s alpha coefficient with 29 items in each scale (excluding items with zero variance) was 0.77 for F1 and 0.79 for F2. The difference between F1 and F2 was not significant (t=0.36, df: 212, p<0.72; F1: 24.10±3.65, F2: 24.15±3.70). 

**Figure 1:**
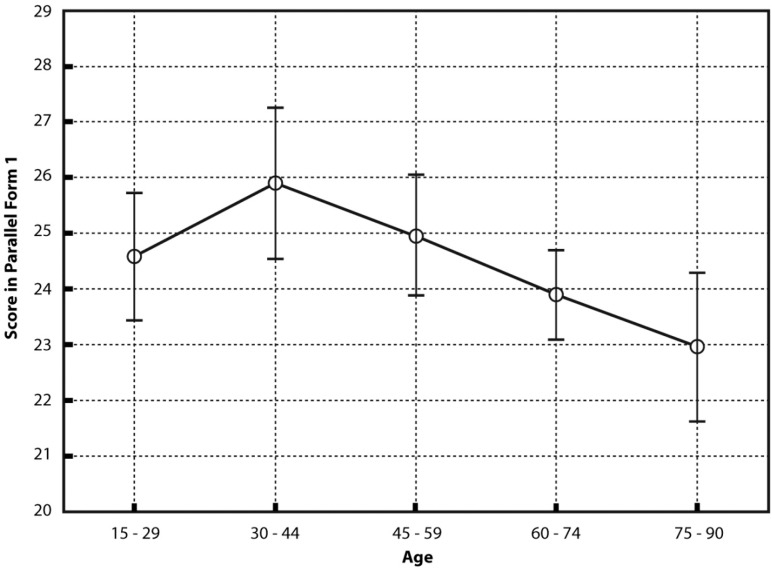
Age effect in a gender × age × education ANOVA on the sponta neous performance of the abbreviated scale (form 1). LS means effective hypothesis decomposition. Vertical bars denote 0.95 confidence intervals.

The three-way ANOVA for demographic variables indicated a significant main effect for age in both forms (F1: F(4,193)=2.96, p=0.02 [see Figure 1]; F2: F(4,193)=2.70, p=0.03) and non-significant effects for the rest of the factors or interactions between factors in either of the forms (F1: gender, education, and gender × education (all F(1,193)≤2.07, p≥0.15); gender × age, age × education, and gender × age × education (all F(4,193)≤1.87, p≥0,12)); (F2: gender, education, and gender × education (all F(1,193)≤2.36, p≥0.13); gender × age, age × education, and gender × age × education (all F(4,193)≤1.99, p≥0,10)). [Results for the initial CNT were very similar].

Just for descriptive purposes, and considering the three CNT, Bonferroni’s post-hoc test indicated that comparisons between age ranges were significant only for women aged 30-44 years with second level of education, who were the participants with the best performance, versus women aged 60-74 and 75-90 years with first level of education, who were the participants with the worst performance. 

We observed a CCI of 0.83 for test-retest and inter-rater reliability for both F1 and F2, without significant differences between the two measures. Indices for test-retest reliability (with an inter-test interval of 61.59±27.85 days): F1: t=1.64, df: 36, p<0.12, Test: 24.76±3.27, Retest: 25.27±3.62; F2: t=1.83, df: 36, p<0.08, Test: 24.62±3.21, Retest: 25.22±3.79. Indices for inter-rater reliability: F1: t=0.80, df: 36, p<0.43, Rater 1: 24.76±3.27, Rater 2: 24.51±3.31; F2: t = 0.46, df: 36, p<0.65, Rater 1: 24.62±3.21, Rater 2: 24.49±3.09. 

**Figure 2:**
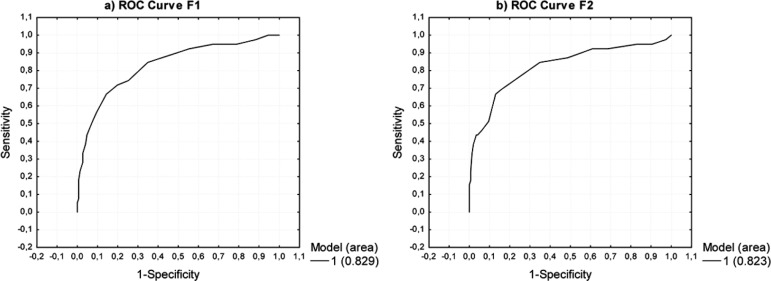
Receiver Operating Characteristic (ROC) curves for the abbreviated scales: Form 1 (F1) and Form 2 (F2). F1 was selected for a computer-aided confrontation-naming evaluation (CACNE). Note: the area under the ROC curve for the initial scale from which F1 and F2 were modeled was 0.839.

The optimal cut-off points for the scales are shown in Table 4 and the ROC curves for the abbreviated scales are depicted in Figure 2. The sensitivity of the initial scale to differentiate non-LD vs. LD was 77% (30/39 for LD) and the specificity was 78% (114/146 for non-LD). The sensitivity of F1 was 74% (29/39 for LD) and the specificity 75% (110/146 for non-LD). F2 had better sensitivity, but lower specificity regarding non-LD because 85% of LD (33/39) scored ≤ the cut-off point, whereas 66% (96/146) scored> the cut-off point. It was not possible to find a model with a more uniform frequency distribution between sensitivity and specificity for F2. 

**Table 4:** Frequency by group according to cut-off points of ROC curves in spontaneous-performance total-score of the three scales.

Group	Initial scale			F1			F2		Total
	Cut-off point: 44			Cut-off point: 22			Cut-off point: 22		
	≤	>		≤	>		≤	>	
LD	30 (77%)	9 (23%)		29 (74%)	10 (26%)		33 (85%)	6 (15%)	39
RD	17 (42%)	23 (58%)		19 (48%)	21 (52%)		23 (58%)	17 (42%)	40
HP	15 (14%)	91 (86%)		17 (16%)	89 (84%)		27 (26%)	79 (74%)	106
Total	62	123		65	120		83	102	185

LD: patients with left hemisphere damage; RD: patients with right hemisphere damage; HP: healthy participants; F1: parallel form 1; F2: parallel form 2. Percentages of row counts are shown. Initial scale: Chi^2^=52.27; df: 2; p<0.0001. F1: Chi^2^=45.97; df: 2; p<0.0001. F2: Chi^2^=43.61; df: 2; p<0.0001. Chi^2^: Chi-square statistics; df: degrees of freedom.

In the matched samples, descriptive data of the total scores for the initial scale, as well as for F1 and F2 were 45.20±8.61; 22.72±4.55; and 22.48±4.37, respectively. The difference between F1 and F2 was not significant (t=1.41, df: 184, p<0.17).

Table 4 shows the frequency distribution according to the cut-off points of the ROC curves. The difference between LD and HP was more relevant, since most LD scored ≤ the cut-off point, whereas most HP scored > the cut-off point. By contrast, the frequency distribution for RD was more uniform since most of these patients scored between 40% and 60%of the cut-off point.

With the exception of maybe internal consistency, F1 was selected for the CACNE in view of fulfilling most of the criteria required for this purpose.

CACNE introduction, instructions, correct words, and translation (from Spanish to English) are shown in Appendix 1. To obtain CACNE material and procedure send an e-mail to nsvigliecca@gmail.com.

## DISCUSSION

In the present study, we validated three CNT versions, according to side of brain injury. Specifically, we abbreviated the initial CNT from 60 to 30 items, by developing two parallel forms from this test and then selecting one of them. We verified the reliability and validity of the selected CNT and how it was influenced by demographic variables. By evaluating score on spontaneous performance, we demonstrated test-retest and inter-rater reliability, internal consistency, as well as its validity for detecting patients with lesions of the dominant hemisphere. Only a significant effect of age in the present sample of HP was observed in the selected CNT. Results were similar for the parallel form. Also, using the selected CNT, we developed the CACNE, in which both spontaneous performance and cued performance were designed to detect anomia in interaction with environmental interventions. 

The present results also showed that the inconsistencies observed in CNT studies regarding either the involvement of the dominant hemisphere, or the detection of anomia in certain disorders,^7^^-^ 13 ^,^ 46 were probably due to the presence of anomia in almost 50% of the patients with lesions to the right hemisphere. Thus, the CNT administered to patients with bilateral damage, which had undergone most validity studies, are more likely to have validation problems because these patients are usually compromised across a range of processes that affect naming.^4^^,^ 46

Considering aging and bilateral damage, semantic dementia, which has been studied to reveal the individual roles of left versus right ATL in semantic function, can be asymmetric in early cases; however, the disease is inherently bilateral and it is thus hard to infer a unilateral model for this function.^13^ According to Mesulam et al.,^26^ semantic memory may not represent a unitary function that can be localized but, instead, the collective and interactive contributions of more fundamental networks, each of which contains modality-selective synaptic hierarchies and domain-specific transmodal hubs.^26^

Taking into account both the probable influence of the type of inputs and the two ATLs on the representation of semantic knowledge,^13^ in the present study we observed a significant deficit in naming for LD, despite using nonverbal inputs. This finding is consistent with the hypothesis that the left ATL has a more prominent role in word retrieval, which in turn is related with verbal outputs and the frontal lobe.^13^ Although we tried to exclude patients with visual-perception difficulties, the influence of the two hemispheres on CNT performance was evidenced not only by the presence of anomia in almost 50% of RD, but also in most LD, particularly when the two groups of patients were compared with HP. This finding is also in line with the observations of Rice et al.^13^ In their study, the right TLE group produced slower and less accurate naming responses compared with the control group, but not to the same level of the left TLE group, suggesting that this hemispheric specialization for semantic memory is graded rather than absolute.

Accordingly, Mesulam et al.^26^ affirmed that recent investigations of semantic dementia and patients with unilateral anterior temporal lesions have revealed functional asymmetries in the contributions of each anterior temporal lobe to verbal versus non-verbal processing domains. Such asymmetrical domain selectivity would presumably constitute a deviation from strict amodality,^26^ as pointed out by Rice et al.,^13^ based on data reported from patients with semantic dementia, functional neuroimaging, and cortical-grid neurophysiological investigations. In the view of Mesulam et al.,^26^ temporal lobe lesions might therefore appear to generate domain-independent amodal impairments that equally disrupt verbal and non-verbal components of semantic memory, only if they are sufficiently large and bilateral to include the language network together with the inferotemporal/fusiform object recognition network (see also studies).^12^^,^ 27

Considering the objectives of the present study, in which only unilateral lesions were assessed, and comparing present findings with previous research by this laboratory,^14^ the effect of demographic variables was reduced. However, in the present study, participants older than 60 years of age were predominantly considered for recruitment, which may have influenced results. The additional items incorporated to the extended CNT, which were addressed to avoid demographic differences, may have also influenced results. 

Overall, the three CNT versions in the present study are quantitative measures which are highly sensitive to procedural changes (see paper).^46^ Therefore, different approaches, items, and samples produce different results, in particular when non-representative recruitment is involved or a wide range of difficulty among items is available.^14^ Nonetheless, such quantitative measures allow more possibilities to manage confounding factors and improve psychometric properties in view of the large number of items involved. For example, the development of two parallel forms as a way to abbreviate the initial scale and select one of the forms according to the study objectives, offers the possibility to identify the intended scale as the better of the two options, thus guaranteeing that either of the options represents (to a large extent) the initial scale. In addition, a CNT with culture-free visual stimuli is valuable because it allows the verbal component of the administration protocol to be adapted to different languages.

Limitations of the study: The current proposal requires that the objectives examined here be verified for the researchers’ particular approach and sample. For example, it is likely that increasing performance in older women with first level of education produces an increase in performance in younger women with higher levels of education. [Note: descriptive data for non-significant results of the present initial scale were very similar to the previously used version,^14^ except for the age range of 30 to 44 years in which women had a better performance than men for the second level of education (results available on request)]. Given we did not report lesion size because of variability of the lesions, it is worth mentioning that, when the location of the lesion is known, naming performance generally declines with lesion size among patients with the same etiology (see article^22^ and its implications for laterality).

## APPENDIX 1

CACNE sections: introduction, instructions, translation, and list of correct words.

**Table:**

**A) INTRODUCTION** In the present study, picture recognition matched with its corresponding name is considered a language indicator of semantic memory. As a result, we believed that spontaneous performance and the semantically cued performance should not be given the same score^28^^-^ 30 since, in the former case, only a visual stimulus is enough to trigger the entire process of correctly remembering the name while, in the second case, the interviewer's help is required to favor semantic (visual) recognition. Instead, we intended the CACNE to have three cycles scored differently according to the interviewer's interventions (i.e. the more attempts in remembering a name, the more performance facilitation, thus the lower the score (see below)). Moreover, from the initial 30 items, and during cued performance, each interviewee has his/her own list of difficult items visually presented with auditory cues. We propose the use of repetition priming to gradually select the items with anomia as a function of their difficulty to be remembered by each interviewee. Specifically, in the first cycle we evaluate spontaneous performance and, in the second and third cycles, we evaluate cued performance by only showing those stimuli that could not be remembered in the previous cycles. The procedure was designed based on the following premise: any previous effort that a person makes to place a perceived object into its corresponding semantic and phonological family of representations activates a memory network, which will subsequently facilitate the recall of the object name. Therefore, a semantic or phonemic cue, along with a new presentation of the object, will act like repetition (direct) priming because the same object is presented in complementary ways. Hamberger^9^ has suggested that the auditory presentation of the stimulus is more sensitive than the visual one for detecting anomia. Additionally, in the process of naming, which implicitly includes the process of language evolution,^3^ the phonemic/phonological information is considered to be the second step after picture recognition or semantic retrieval.^11^^,^ 31 ^,^ 36 Therefore, in the CACNE, after verifying and scoring with the maximum value spontaneous recall, using just visual stimuli, we successively used the semantic and phonemic cues, with decreasing scores, as complementary auditory presentations. Since the phonemic cue not only induces the recall of the name more explicitly in language terms, but has also proven to be more effective than the semantic cue to improve naming accuracy,^31^ then in the CACNE, phonemic cued performance is scored with the minimum value. [Note: From now on, we indistinctly use the terms 'phonemic' and 'phonetic' as well as the terms 'cues' and 'helps'.] Anomia can be inferred in the CACNE from the successive results described in each cycle, including the number of correct responses and the type of errors according to the provided cues or helps. The scores in each cycle reflect both the interviewee's accuracy and all the interviewer's interventions, together with their respective weights (the effect of priming can be inferred from the results obtained in the second and third cycles). Consequently, in one session we may observe not only if the person failed to remember a name, but also if some of the processes the brain uses to successively retrieve that name were successful; i.e., if the interviewee recalls the name after anomia, this can be taken as an indication that the name was in the interviewee's repertory and that the link between the picture and the name had probably been weakened for the reasons (or results) explained in the evaluation. Considering the limiting difficulties that may be found in public hospitals, the CACNE was designed to be administered offline, using Flash Player.	
**B) INSTRUCTIONS** **b1) Instructions for the Interviewer** There are three cycles in the evaluation. Each cycle is specified in the title and in the upper left corner of the screen with a red circle, which is highlighted within the other two cycles. During the first cycle, the interviewer must register, in the bottom left corner of the screen, either if the answer was right or, otherwise, the type of cue needed in the next cycle as a function of the type of error. Specifically: in this corner there are four options, the right answer and three types of cues or helps, which have to be administered in the next cycles according to the following criteria: a) If the interviewee did not visually recognize the picture (if he/she said: "I do not know" or if the picture was misidentified or incorrectly described), a ***semantic cue*** should be offered in the second cycle, by briefly describing the object in simple words; b) If the interviewee said a similar name, i.e. a name of the same family of objects or a name with similar pronunciation, but not the exact name, ***a better answer*** should be requested in the second cycle, up to a maximum of two requests; c) If the interviewee did recognize the picture by correctly describing its meaning, but the name was not remembered (anomia), a ***phonetic cue*** should be offered in the third cycle, up to a maximum of two requests if the name has more than two syllables (the first request by saying the initial sound of the name, the second request by saying the two initial sounds). This last cue will also be offered if the interviewee failed with the previous cues. [Note: Each type of selected cue is automatically recognized by the program to be administered in the corresponding cycle]. In the second and third cycles, the interviewer will only register if the final answer was right or wrong.	
Additional Comments: 1) Interviewee's self-corrections are not counted as errors. 2) Wrong answers, which are registered verbatim in the three cycles, include picture descriptions. 3) Words or expressions that are distorted or not related to the picture represent wrong answers or descriptions; the offered help will depend on the cycle in which they occur. 4) Errors in the third cycle are taken as indicators that, under the current conditions, those names are unknown. 5) When the last picture of each cycle has been administered, interviewers can examine the "Partial Results" in the first and second cycles, or the "Final Results" (including the partial ones) in the third cycle. 6) Interviewers should never press 'New Test' unless they want to start all over again from the first picture. 7) Although each researcher will have prepared in advance his/her own list of semantic cues according to the particular sample recruited, when the partial results are observed, interviewers will have the opportunity to think about the cues for the wrong answers because the time record stops at that stage. 8) The list of correct names shown in part (d) of this table is tentative, based on our experience, but researchers will be able to adapt it to their language or cultures, including the corresponding cues.	
**b2) Instructions for the Interviewee** Initial or Spontaneous Performance *I am going to show you some pictures and I want you to tell me their names. If you cannot say the exact name, give your best answer or say "I don't know" to go to the next item. Later you will be able to tell me what you know about that object.* *What's this?* Cued Performance *Now you can tell me the name or what you know about those pictures that you couldn't remember the name of before. I am going to help you.* *What's this?*	
**C) TRANSLATION OF THE INSTRUCTIONS AND TERMS USED IN CACNE** [Note: Spanish terms in italic, English terms in bold. see Fig. 2] Evaluación de Nombres, por Confrontación, Asistida por Computadora (ENCAC) **Computer-Aided Confrontation-Naming Evaluation (CACNE)** *Test de denominación - Ciclo 1: Desempeño espontáneo/* **Naming test** **- Cycle 1: Spontaneous performance** *Tenga la lista de palabras correctas* / **Have the list of correct words** *Escriba textualmente las palabras erradas* / **Write the wrong words verbatim** [*Confirme las actividades para continuar...* / **Confirm the activities to continue...]** 1: *Desempeño espontáneo* / **1: Spontaneous performance** 2: *Desempeño con 1º ayuda* / **2: Performance with 1st help** 3: *Desempeño con 2º ayuda* / **3: Performance with 2nd help** *Bien* / **Good** *Semántica* / **Semantic** *Mejor Resp.*/ **Better Answer** *Fonética* / **Phonetic** [*Seleccione una opción...* / **Choose an option...**] *Nuevo Test* / **New Test** [*¿Desea interrumpir el test en curso? SI NO*/ **Do you want to interrupt the test in progress? YES NO]** *Test de denominación* / **Naming test** *Nuevo ciclo* / **New cycle** *Test de denominación - Ciclo 2: Desempeño con 1º ayuda /* **Naming test - Cycle 2: Performance with 1st help** *Ofrezca una ayuda semántica* / **Offer a semantic help** *Pida una mejor respuesta* / **Ask for a better answer** *Test de denominación - Ciclo 3: Desempeño con 2º ayuda /* **Naming test - Cycle 3: Performance with 2nd help** *Ofrezca una ayuda fonética* / **Offer a phonetic help** *Resultados Parciales* / **Partial Results** *Resultados Finales* / **Final Results** *Test de denominación* - *Resultados Finales/* **Naming test - Final Results** *Tiempo Empleado: minutos (mm) y segundos (ss) /* **Completion Time: minutes (mm) and seconds (ss)** *Puntaje Total* / **Total Score** *Ciclo 1: Desempeño espontáneo* / **Cycle 1: Spontaneous performance** *Tiempo (mm:ss)* / Time (mm:ss) *Figuras Puntaje Correctas Semántica Mejor Resp. Fonética /* **Figures Score Correct Semantic Better Answer Phonetic** *Ciclo 2: Desempeño con ayuda* / **Cycle 2: Performance with help** *Tiempo (mm:ss)* / Time (mm:ss) Figuras Puntaje Correctas Semánticas Correctas Mejor Resp. Erróneas **Figures Score Correct Semantic Correct Better Answer Wrong** *Ciclo 3: Desempeño con ayuda* / **Cycle 3: Performance with help** *Tiempo (mm:ss)* / **Time (mm:ss)** *Figuras Puntaje Correctas Erróneas* **Figures Score Correct Wrong**	
**D) LIST OF CORRECT WORDS IN OUR SAMPLE OF SPANISH SPEAKERS AND TRANSLATION** [Note: Items are in order of difficulty]	
**Spanish** 1) reloj 2) teléfono 3) libro 4) cama 5) silla 6) sobre (carta) 7) rastrillo 8) piano 9) molino -de viento- 10) cigarrillo 11) lámpara (velador) 12) helicóptero 13) cepillo de dientes 14) pulpo 15) jeringa (inyección) 16) dado 17) cafetera 18) grúa (guinche) 19) caballete 20) candelabro (candelero) 21) verja (cerca/o) 22) matafuego (extintor, extinguidor) 23) guadaña 24) cono 25) puntilla (guarda) 26) clave de sol 27) yunque (bigornia) 28) cilindro 29) gaita 30) diapasón	**English** 1) clock 2) telephone 3) book 4) bed 5) chair 6) envelope (letter) 7) rake 8) piano 9) windmill 10) cigarette 11) lamp 12) helicopter 13) toothbrush 14) octopus 15) syringe (injection) 16) die (dice) 17) coffee pot 18) crane 19) sawhorse (trestle) 20) candelabrum (candlestick) 21) fence 22) fire extinguisher 23) scythe 24) cone 25) lace (lace edging) 26) treble clef 27) anvil 28) cylinder 29) bagpipe 30) tuning fork
