# Errata

**DOI:** 10.1590/1980-57642020dn14-010016

**Published:** 2020

In the paper Vigliecca NS, Voos JA – Remembering a name: neuropsychological validity studies and a computer proposal for detection of anomia, DOI number 10.1590/1980-57642018dn13-040013, published in the journal Dement Neuropsychol. 2019;13(4):450-462, in the page 450.

Where it reads:

1. Servicio de Neurología y Neurocirurgía del Hospital Córdoba, Argentina.

Read:

1. Instituto de Humanidades, Consejo Nacional de Investigaciones Científicas y Técnicas de la Argentina (IDH-CONICET), Universidad Nacional de Córdoba (UNC); Servicio de Neurología y Neurocirugía del Hospital Córdoba, Argentina.

